# A Ligamentous Agony: Median Arcuate Ligament Syndrome as an Under-Recognized Cause of Abdominal Pain

**DOI:** 10.7759/cureus.8865

**Published:** 2020-06-27

**Authors:** Dharmini Manogna, Atul Gupta, Mysore Seetharaman

**Affiliations:** 1 Internal Medicine, Rochester General Hospital, Rochester, USA; 2 Radiology, Rochester General Hospital, Rochester, USA

**Keywords:** median arcuate ligament syndrome, chronic abdominal pain, celiac artery compression

## Abstract

Median arcuate ligament (MAL) syndrome (MALS) is a rare clinical entity characterized by chronic abdominal pain resulting from compression of the celiac artery by the MAL. We present a case of MALS with imaging evidence of anterior compression of the celiac artery on expiration, which was relieved on inspiration. A 33-year-old woman presented with intermittent upper abdominal pain since three months. The pain was associated with nausea, abdominal bloating and diarrhea. Physical examination revealed a palpable abdominal aorta with no bruit. Abdominal sonogram, upper and lower gastrointestinal endoscopies, celiac disease screening, clostridium difficile toxin assays and hepatobiliary iminodiacetic acid scan were all normal. Computerized tomography angiogram (CTA) revealed subtle narrowing at the origin of the celiac artery without any atherosclerosis or calcification. Lateral aortic angiography showed anterior impression on the celiac artery at its origin on expiration. The constriction was relieved on inspiratory film - findings most consistent with MALS. MALS is rare, typically presenting with non-specific symptoms including nausea, vomiting, chronic post-prandial abdominal pain or unintentional weight loss. Angiography with respiratory maneuvers remains the diagnostic standard. However, non-invasive vascular imaging during both phases of respiration can be considered as an initial diagnostic test. The primary goal of therapy is celiac artery decompression with the additional objective of neuronolysis of the celiac ganglion. Our case highlights that MALS should be considered as a differential diagnosis in chronic, recurrent abdominal pain, particularly with an unrevealing initial evaluation. Evidence of celiac artery compression on vascular imaging, with characteristic respiratory variation, is highly suggestive of MALS.

## Introduction

Median arcuate ligament (MAL) syndrome (MALS) is an uncommon clinical entity caused by compression of the celiac artery by the MAL. The MAL is a band of fibrous tissue that connects the diaphragmatic crura surrounding the aortic hiatus anteriorly. It can manifest as an amalgamation of vague symptoms including abdominal pain, early satiety and food regurgitation, which are not pathognomonic to MALS [1]. Therefore, it is usually a diagnosis of exclusion. This often poses a diagnostic challenge, with most patients undergoing extensive evaluation with unrevealing findings. This has led to much debate about the existence of the syndrome, diagnostic features and consequently, appropriate therapeutic modality in this setting [2]. Computed tomographic angiography (CTA) is coming to prove instrumental in the diagnosis of this infrequent disease. A distinctive, focal hook-like narrowing of the celiac axis on CTA is strongly supportive [3]. We herein report a case of MALS with classic imaging findings of respiratory variation in vascular compression, which aided in making this rare diagnosis.

## Case presentation

A 33-year-old woman was evaluated for upper abdominal pain of three months’ duration. The pain was intermittent, occasionally postprandial and was associated with an unintentional 12-pound weight loss over the same period. She also indicated that the pain worsened with exercise and found some relief in the knee chest position. She noted associated nausea, abdominal bloating and diarrhea. There was no history of fever, chills, hematemesis, melena or excessive use of non-steroidal analgesics. Furthermore, she denied travel and exposure to sick contacts. Past medical history was unremarkable. Alcohol and drug use was denied. Physical examination was notable for a palpable abdominal aorta with no bruit.

**Figure 1:**
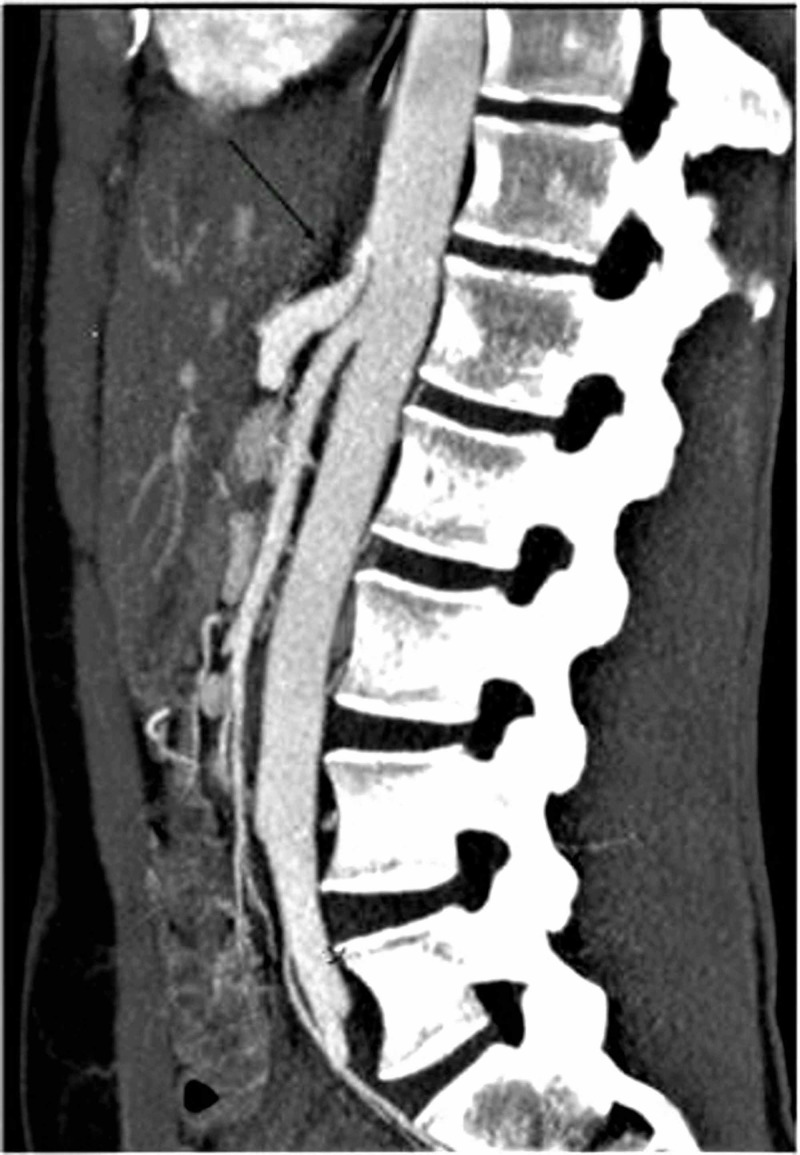
Lateral computerized tomography angiogram The angiogram showing mild anterior impression on the celiac artery at its origin.

**Figure 2:**
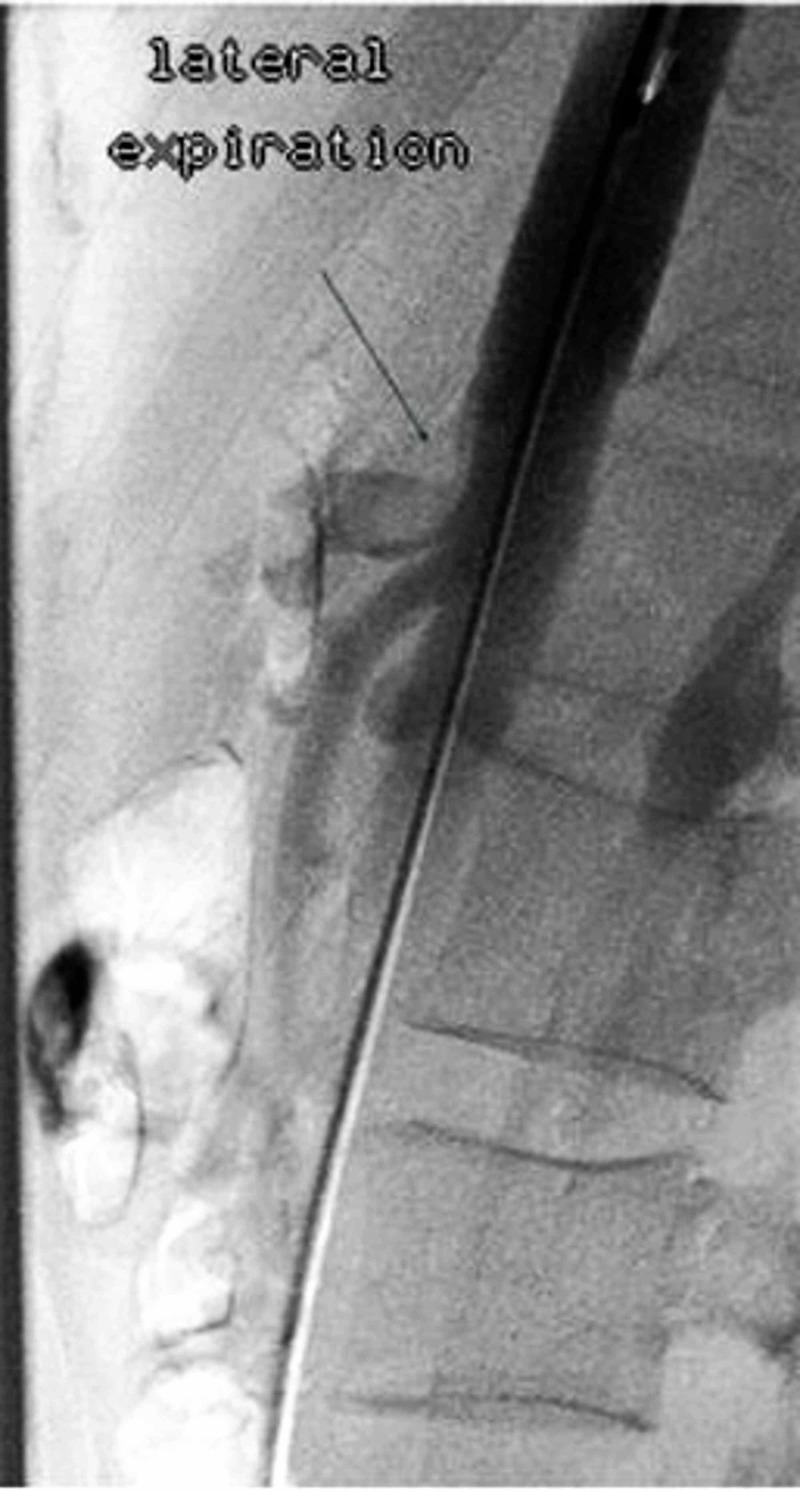
Expiratory lateral aortic angiogram The angiogram on expiration showing anterior impression on the celiac artery and constriction at its origin.

**Figure 3:**
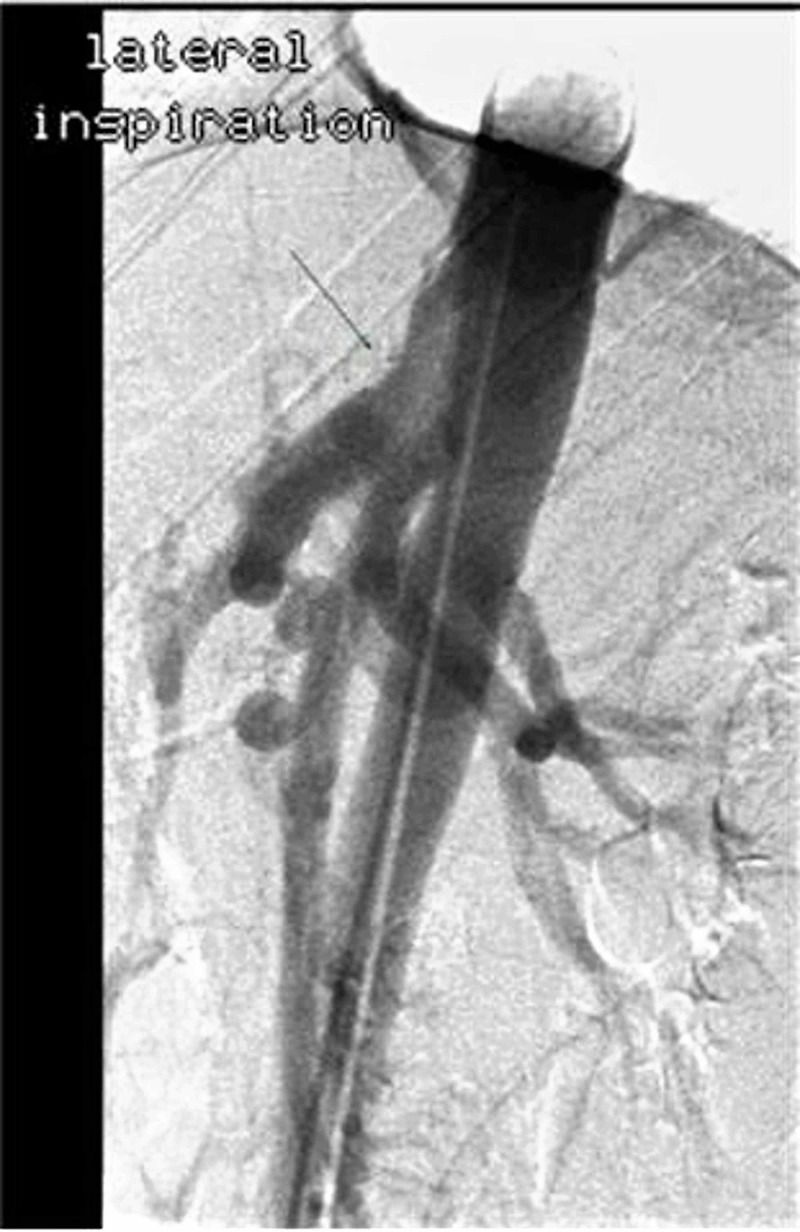
Inspiratory lateral aortic angiogram The angiogram on inspiration depicting release of the anterior impression and constriction of the celiac artery.

Clinical work-up consisted of a normal abdominal sonogram and unremarkable upper and lower gastrointestinal endoscopies. Celiac disease screening, clostridium difficile toxin assay and hepatobiliary iminodiacetic acid scan were unrevealing. Persistent abdominal symptoms and weight loss prompted search for a vascular etiology. CTA revealed an eccentric narrowing on the anterior surface of the celiac trunk without any atherosclerosis or calcification, warranting conventional catheter angiography under respiratory maneuvers (Figure 1). The lateral catheter angiogram was remarkable for anterior impression on the celiac trunk at its origin on expiration (Figure 2). The constriction was relieved on inspiratory film - findings most consistent with MALS (Figure 3). Surgical intervention was recommended to our patient. However, due to social and domestic reasons, she opted to consider laparoscopic celiac artery decompression at a later date.

## Discussion

The pathophysiology of MALS is unclear and is also controversial because a considerable proportion of patients may remain asymptomatic. The anterior MAL is a fibrous band that connects the diaphragmatic crura anteriorly and surrounds the aortic hiatus. Patients with a high origin of the celiac artery or lower insertion of the diaphragm are vulnerable to celiac artery compression. This compression has been demonstrated to be relieved by inspiratory caudal movement of the MAL, while the reverse is true in expiration.

The diagnosis of MALS is challenging and is one of exclusion; as a result, typically these patients undergo an extensive evaluation to rule out celiac disease, chronic gallbladder disease, infectious causes and malignancy. These etiologies were considered in our patient and were ruled out after a comprehensive work-up. Clinically, the syndrome is more frequently seen in young and middle-aged female patients with a 4:1 female preponderance [1]. Symptoms are usually non-specific and variable, including nausea, vomiting, chronic post prandial abdominal pain or unintentional weight loss. Physical examination may reveal a mid-epigastric abdominal bruit, which varies with respiration. Duplex abdominal ultrasonography during both phases of respiration can be considered as an initial study to assess vascular compromise with a great degree of sensitivity and specificity [2]. CTA and magnetic resonance (MR) angiography are additional non-invasive diagnostic imaging studies that have the added advantage of three-dimensional reconstruction of images. Through direct visualization of the celiac artery compression during CTA, we eliminated other concomitant abdominal lesions such as atherosclerosis as the causative factor in our patient. Angiography with respiratory maneuvers remains the diagnostic standard [3]. Gastric tonometry measures intramucosal and intraluminal partial pressure of carbon dioxide (PaCO_2_) levels, and is an indicator of gastric ischemia. It has utility not only for diagnosis but also for follow-up after celiac trunk decompression [4]. Celiac plexus neuronolysis has been attempted with the understanding that inflammation and compression of the celiac plexus could, in part, be responsible for the clinical symptoms. Kim et al. have suggested an algorithmic approach to diagnosis of MALS [5].

The primary goal of therapy for MALS is celiac artery decompression with the additional objective of neuronolysis of the celiac ganglion. The traditional approach had been open decompression involving removal of the celiac plexus and MAL [6]. Open surgical options have included exploratory laparotomy and decompression alone, decompression with celiac dilatation and decompression with reconstruction and bypass of the stenosed celiac artery. Laparoscopic release of celiac artery compression has gained ground and is being increasingly recognized as an effective alternative to the traditional open approach [7]. Advantages of laparoscopic approach include smaller incision, decreased postoperative morbidity, shorter hospital stay and quicker enteric feeding. However, disadvantages are possibility of incomplete release, intra-abdominal hemorrhage, aortic injury and the potential for conversion to open approach in the case of fixed celiac artery stenosis. Adjunctive therapy in the management of MALS includes arteriography and percutaneous transluminal angioplasty with balloon expandable stent, particularly in patients with residual symptoms or persistent postoperative morbidity. The algorithmic approach to the management of MALS is very well described by Kim et al. [5]. Robotic assisted division of MALS is one of the emerging armamentarium for the division of MALS and neuronolysis [8].

## Conclusions

Our experience with this case demonstrates a classic imaging finding in the background of highly suggestive symptoms, which strongly support the existence of MALS. CTA can be valuable in clinching this diagnosis. It should be considered after an initially unremarkable gastroenterological work-up, when the presentation is redolent of MALS. This can lead to early diagnosis and surgical management, which can often result in complete resolution of symptoms.

## References

[1] Trinidad-Hernandez M, Keith P, Habib I, White JV (2006). Reversible gastroparesis: functional documentation of celiac axis compression syndrome and postoperative improvement. Am Surg.

[2] Gruber H, Loizides A, Peer S, Gruber I (2012). Ultrasound of the median arcuate ligament syndrome: a new approach to diagnosis. Medical Ultrason.

[3] Horton KM, Talamini MA, Fishman EK (2005). Median arcuate ligament syndrome: evaluation with CT angiography. Radiographics.

[4] Mensink PB, van Petersen AS, Kolkman JJ, Otte JA, Huisman AB, Geelkerken RH (2006). Gastric exercise tonometry: the key investigation in patients with suspected celiac artery compression syndrome. J Vasc Surg.

[5] Kim EN, Lamb K, Relles D, Moudgill N, DiMuzio PJ, Eisenberg JA (2016). Median arcuate ligament syndrome—review of this rare disease. JAMA Surg.

[6] Harjola PT (1963). A rare obstruction of the coeliac artery. Report of a case. Ann Chir Gynaecol Fenn.

[7] Tulloch AW, Jimenez JC, Lawrence PF (2010). Laparoscopic versus open celiac ganglionectomy in patients with median arcuate ligament syndrome. J Vasc Surg.

[8] Jaik NP, Stawicki SP, Weger NS, Lukaszczyk JJ (2007). Celiac artery compression syndrome: successful utilization of robotic-assisted laparoscopic approach. J Gastrointestin Liv Dis.

